# The Comparative Effects of Supplementing Protease Combined with Carbohydrase Enzymes on the Performance and Egg n-3 Deposition of Laying Hens Fed with Corn-Flaxseed or Wheat-Flaxseed Diets

**DOI:** 10.3390/ani13223510

**Published:** 2023-11-14

**Authors:** Jinyi Wan, Muhammad Suhaib Shahid, Jianmin Yuan

**Affiliations:** 1 College of Veterinary Medicine, China Agricultural University, Beijing 100193, China; duet4536251@163.com; 2 State Key Laboratory of Animal Nutrition, College of Animal Science and Technology, China Agricultural University, Beijing 100193, China; suhaib_shahid@yahoo.com

**Keywords:** flaxseed, protease, carbohydrase enzymes, fatty acid, egg, fatty acid transport, layer hens

## Abstract

**Simple Summary:**

A wheat-flaxseed diet (WFD) and a corn-flaxseed diet (CFD) were provided with three different proteases and multi-carbohydrase enzymes added to compare their effects on laying hen performance, egg n-3 deposition, and fatty acid transporter genes in laying hens. The WFD diets with enzyme B produced the heaviest eggs in the 9–10th week. The WFDs had more total n-3 (468.22 mg/egg) compared to the CFDs (397.90 mg/egg). Addition of enzyme C (464.90 mg/egg) deposited more total n-3 in eggs compared to enzymes A and B (411.89 and 422.42 mg/egg). The WFD with enzyme C had higher docosahexaenoic acid, and a reduced n-6:n-3 ratio in the egg yolks compared to the CFD. The wheat-flaxseed diets showed higher expression of liver fatty acid binding protein (*p* = 0.006), fatty acid desaturase 1, elongase-2 (ELOV-2), and fatty acid transport protein-1 compared with the CFD. The WFD with enzyme C contributed to better performance, enrichment of eggs with n-3 and DHA, and upregulation of fatty acid transporter genes.

**Abstract:**

Flaxseed contains huge quantities of anti-nutritional factors (ANFs), which reduce the performance of livestock. Three different protease and multi-carbohydrase enzymes were included in wheat-flaxseed diets (WFD) and corn-flaxseed diets (CFD) to compare their effects on performance, egg n-3 deposition, and fatty acid transporter genes in laying hens. A total of 540, twenty-week-old, Nongda-3 laying hens (DW brown × Hy-line white) were randomly assigned to six dietary groups, including 10% WFD or 10% CFD plus (i) supplemental enzyme A (alkaline protease 40,000 and neutral protease 10,000 (U/g)), (ii) enzyme B (alkaline protease 40,000, neutral protease 10,000, and cellulase 4000 (U/g)), or iii) enzyme C (neutral protease 10,000, xylanase 35,000, β-mannanase 1500, β-glucanase 2000, cellulose 500, amylase 100, and pectinase 10,000 (U/g)). An interaction (*p* < 0.05) was found for egg mass, hen day of egg production, and feed conversion ratio on the 9–10th week of the experiment. The WFD with enzyme B was associated with the highest egg weight in the 9–10th week. The deposition of total n-3 was superior with WFD (468.22 mg/egg) compared to CFD (397.90 mg/egg), while addition of enzyme C (464.90 mg/egg) resulted in the deposition of more total n-3 compared to enzymes A and B (411.89 and 422.42 mg/egg). The WFD and enzyme C significantly (*p* < 0.001) enhanced docosahexaenoic acid (DHA) and reduced the n-6:n-3 ratio in egg yolk compared to the CFD. The hepatic mRNA expression of liver fatty acid binding protein (L-FABP) (*p* = 0.006), fatty acid desaturase 1 (FADS-1) (*p* < 0.001), elongase-2 (ELOV-2) (*p* < 0.001), fatty acid transport protein-1 (FATP1) (*p* < 0.001), and the intestinal mRNA expression of FATP and FABP genes were increased with WFD compared to CFD. In conclusion, WFD with enzyme C is favorable for optimal performance, results in the deposition of more n-3 and DHA, and increases the expression of fatty acid transporter genes, which helps in n-3 transport.

## 1. Introduction

The n-3 polyunsaturated fatty acids (PUFAs) are beneficial to human health, and poultry products rich in n-3 are popular in the market. Flaxseed is the best source of alpha-linolenic acid (ALA; C18:3 n-3 or omega-3 fatty acid) and is widely used to produce n-3 PUFAs in poultry meat and eggs [1,2]. However, flaxseed contains high levels of anti-nutritional factors (ANFs), such as mucilage from the hull, non-starch polysaccharides (NSPs), cyanogenic glycosides, dipeptide linatine (vitamin B6 antagonist), trypsin inhibitors, and phytic acid [3,4], which reduce the secretion of pancreatic enzymes, decrease the digestibility of the feed, increase the viscosity of the intestinal digesta [2,5], change the intestinal flora [6], and lead to acute colonic mucosal damage and inflammation [7]. Moreover, higher levels of flaxseed, and or long-term flaxseed feeding, decrease the performance of laying hens or broiler chickens [1,8]. To overcome the adverse effects of ANFs, mechanical processing techniques, such as grinding, pressing, refining, and heating, are commonly employed [9]. However, processing the flaxseeds in this manner can cause fatty acid oxidation, fire hazards, and reduce shelf life.

Usually, wheat is combined with flaxseed to produce n-3 eggs due to its lower n-6 PUFAs. The cell wall of wheat also contains anti-nutritional factors in the form of NSPs [10], which are mostly arabinoxylan polymers [11,12]. Enzymes are often added to the diet of chickens to eliminate the adverse effects of ANFs. An in vitro study showed a 37.6% reduction in NSPs when flaxseed was combined with pectinase, cellulase, xylanase, mannanase, glucanase, and galactanase [13]. Supplemental carbohydrase enzymes can improve the morphology of the gastrointestinal tract of laying hens fed flaxseed [14], and enhance the deposition of total n-3 fatty acids in the eggs of laying hens [2]. 

The proteases or proteinases are proteolytic enzymes that can be used to hydrolyze the trypsin inhibitor of flaxseed [5]. A study reported that protease in corn-based diets increases the chyme emptying time and trypsin activity, leading to enhanced energy absorption [15]. However, the impact of proteases on amino acid digestibility is dependent on the product, supplementation level, and diet composition [16,17]. Previous studies have shown that the use of an enzyme blend containing 300 U of cellulase, 3950 U of mannanase, and 5000 U of pectinase significantly reduced the viscosity of chow in broilers fed a flaxseed diet [18]. Research has suggested that multi-carbohydrate enzymes might be more effective in degrading NSP than individual enzymes [10,19].

It is evident that corn contains less (9.0%) total NSP compared with wheat (11.3%) [20]; however, wheat is mostly used to produce n-3 PUFA eggs due to its lower fat content (1.74%) and higher n-3 content (4.16%) compared with corn, which has higher fat content (4.4%) and lower n-3 content (2.15%) [21]. The competition between n-3 fatty acids with n-6 fatty acids on the absorption of n-3 fatty acids is unknown. Moreover, there are multiple anti-nutritional factors in a flaxseed-wheat diet, so protease should be used alone, or combined with other multi-carbohydrase enzymes that can reduce the harmful effects of the ANFs of flaxseed. Therefore, this study was designed to explore which enzyme blend and diet combination is most useful to produce n-3 PUFA eggs, and to investigate whether competition exists in fatty acid absorption and transport between corn more rich in fats and with less C18:3 added with flaxseed rich in C18:3, compared with wheat that contains less fat content combined with flaxseed with higher levels of C18:3.

## 2. Materials and Methods

### 2.1. Animal Ethics

All the animal trials were carried out under the protocol of the Chinese Regulations for Laboratory Animals. The Laboratory Animal Ethical Committee of the China Agricultural University (CAU) approved the experimental animal protocols (AW04110202-1). The experiment was carried out at the CAU experimental base in Zhuozhou, Hebei, China.

### 2.2. Bird Care and Management

A total of 540 Nongda-3 hens, 24 weeks old, were initially weighed and then randomly allocated to six dietary treatments with six replicates in each group. Each replicate had fifteen hens, with separate compartments for three hens in the respective cages. The dimensions of each cage were 0.6 m × 1.35 m × 0.4 m. Hens were kept in an automatically controlled house with average temperature and relative humidity controlled at 22 °C and 53%, respectively. The light period was 16 h. The water was provided ad libitum in nipple drinkers and feed was given according to the laying hens’ standard requirements during the ten-week experiment.

### 2.3. Enzymes Description

Three enzyme combinations were purchased from Asia-Pac (Dongguan, China) Bio-technology Co., Ltd. (Guangdong, China). Enzyme A contained neutral protease 10,000 U/g and alkaline protease 40,000 U/g. Enzyme B contained neutral protease 10,000 U/g, alkaline protease 40,000 U/g, and cellulase 4000 U/g. Enzyme C contained neutral protease 10,000 U/g, xylanase 35,000 U/g, β-mannanase 1500 U/g, β-glucanase 2000 U/g, cellulose 500 U/g, amylase 100 U/g, and pectinase 10,000 U/g. 

### 2.4. Diets Preparation

A 2 × 3 two factorial arrangement was used in the research trial. The two basic diets were based on corn and wheat; each diet was added with 10% whole flaxseed. Each diet was supplemented with 200 g/ton of one of three different multi-carbohydrase enzymes. The diets were formulated to be iso-caloric and iso-nitrogenous and to meet or exceed [22] the requirements for laying hens. All six diets were offered to the laying hens for a period of 70 d (10 weeks) as presented in Table 1.

### 2.5. Flock Performance

The bodyweight gain (BWG), feed intake (FI), and feed conversion ratio (FCR) were documented every two weeks of the experiment. Eggs were collected and weighed daily to calculate the egg mass and egg production. The hen day of egg production (HDEP) was recorded daily. 

### 2.6. Egg Sample Collection for Fatty Acid Analysis

For the fatty acid analysis, 3 eggs from each replicate were selected at the end of the 2nd, 4th, 6th, 8th, and 10th weeks of the experiment. Three egg yolks for every replicate were separated with an egg yolk separator, rolled on filter paper, pooled, freeze-dried at −80 °C, and stored at −80 °C for fatty acid analysis.

### 2.7. Fatty Acid Analysis

The fatty acid methyl esters (FAMEs) of the egg yolks were prepared using direct FAME synthesis [23]. The FAMEs were subjected to gas chromatography-mass spectrometry (SCION-456, Shanghai, China) for fatty acid separation. The column measurements were (30 m × 0.32 mm × 0.25 µm, Zebron ZB-WAXplus, Suzhou, China). The carrier gas used was helium at a flow rate of 1.5 cm 3/min. The column, detector, and injector temperatures were set at 195, 250, and 225 °C, respectively. The FAs were identified and quantified by comparing their retention times with the standard Supelco 37 Component FAME Mix C4-C24 (Sigma-Aldrich, Hamburg, Germany). Undecanoic fatty acid (C11:0) was used as an internal standard to allow the conversion of the relative percentage (%FA/total FA) of each fatty acid in absolute value as mg/egg.

### 2.8. RNA Extraction and Reverse Transcription

At the end of ten weeks, one bird from each replicate was sacrificed. The liver, intestine, and abdominal fat were collected and stored at −80 °C for the analysis of gene expression. The total RNA of the liver samples was extracted using Trizol Reagent (Invitrogen Biotechnology Inc., Carlsbad, CA, USA) according to the manufacturer’s protocol. The gene (Table 2) mRNA expression was measured by an ABI 7500 Real-time PCR system (Applied Biosystems, Foster City, CA, USA), as described by Shin [24]. Real-time PCR was carried out using SYBR Premix Ex Taq (TliRNaseH Plus) (Takara Biotechnology Inc., Osaka, Japan). The reaction volume of the 20 μL mixture contained 10 μL SYBR Premix Ex Taq (TliRNaseH Plus) (2×), 0.4 μL ROX Reference Dye-II (50×), 0.4 μL of each forward and reverse primer (Table 2), 6.8 μL Easy Dilution, and 2 μL cDNA template. The optimized protocol for all the genes was 95 °C for 30 s followed by forty cycles of 95 °C for 5 s and 60 °C for 34 s. All measurements were carried out in triplicate and the average values were obtained. The real-time PCR efficiency for each gene was calculated based on the slope of the cDNA relative standard curve that was formulated using a pooled sample. The specificity of the PCR products was evaluated by analysis of the melting curve. The results for the relative mRNA expression of genes were calculated using the 2^−ΔΔCt^ method [25]. 

### 2.9. Statistical Analysis

A 2-way ANOVA was used as a 2 × 3 factorial arrangement of treatments. Data were analyzed as a completely randomized design by SPSS 20.0 [26]. Post hoc multi-comparisons were applied using Duncan’s test to compare the means of the dietary treatment groups. Significance was set at *p* < 0.05.

## 3. Results

### 3.1. Production Performance

The effects of CFD and WFD with enzymes on the production performances are shown in Table 3. A significant interaction between diets and enzymes was recorded for BWG during 0–2 weeks where a wheat-flaxseed diet with enzyme C showed the highest body weight gain. An interaction was recorded at 3–4 weeks for BWG in which corn-flaxseed with enzyme C showed the highest weight compared to the other groups. The WFD reduced egg weight between 0–2 weeks and 3–4 weeks (*p* < 0.05), hen day egg production from 0–2 weeks (*p* = 0.050), and feed intake between 0–2, 3–4, and 5–6 weeks (*p* < 0.001), compared to CFD. However, no variation was found between the other periods. The enzyme combination significantly (*p* < 0.05) affected the egg mass, HDEP, and FCR at 3–4 weeks. During this period, the egg mass and HDEP were highest, while FCR was the lowest with enzyme A. At 7–8 weeks, there was an interaction (*p* = 0.044) for feed intake, where the corn-flaxseed with enzyme B group of hens consumed more feed.

However, in the 9–10th week, there was an interaction (*p* < 0.05) for the egg mass, HDEP, and FCR. In the 9–10th week, the HDEP was highest for the corn-flaxseed with enzyme C group, and egg mass was highest in the wheat-flaxseed with enzyme B group, while FCR was lowest in the wheat-flaxseed with enzyme B group. In the 10th week, the diet source as well as the enzyme source affected the egg weight, with the wheat-flaxseed with enzyme B group producing the eggs with the highest weight. 

Overall, there was no interaction between the diets and the enzymes on the total egg weight, egg mass, hen day of egg production, body weight gain, and feed conversion ratio, except for feed intake (*p* = 0.047). The CFD with enzyme B group consumed more feed compared to the other groups. However, the enzyme sources affected the total body weight gain (*p* = 0.040), with enzyme C having the highest total BWG compared to the other enzyme combination groups.

### 3.2. Fatty Acid Composition of Egg Yolk (mg/Egg)

The effect of the corn-flax and wheat-flaxseed diets with added enzymes on the egg fatty acid profile of laying hens is presented in Table 4. In the 2nd week (*p* ≤ 0.001) and 10th week (*p* = 0.037) for DHA, there was an interaction between diets and enzymes with the highest values for wheat-flaxseed with enzyme C. On the 4th and 8th weeks, the wheat-flaxseed diet was associated with the maximum (*p* < 0.05) DHA levels compared to the corn-flaxseed diet, while on the 6th week, the wheat-flaxseed diet with enzyme C had significantly (*p* < 0.05) the highest DHA compared to the other diet and enzyme combination groups.

In the 2nd week (*p* = 0.005), 4th week (*p* = 0.046), and 10th week (*p* ≤ 0.001), an interaction was documented for total n-6 between the diets and enzymes, with the highest values for corn-flaxseed and enzyme B (2nd and 10th week) and for enzyme A (4th week). In the 6th week (*p* ≤ 0.001) and the 8th week (*p* = 0.001), the corn-flaxseed diet had higher total n-6 FAs than the wheat-flaxseed diet.

In the 2nd week, the wheat-flaxseed diet (*p* ≤ 0.001) had more total n-3 FAs than the corn-flaxseed diet. In the 4th week, the enzyme sources (*p* ≤ 0.001) were significant for total n-3 FAs, with enzyme C possessing the maximum value. In the 6th week (*p* = 0.001), the wheat-flaxseed diet with enzyme C showed the highest total n-3 FAs. In the 8th week (*p* = 0.001) and the 10th week (*p* = 0.001), for total n-3 FAs, an interaction was noted between the diet and enzyme sources, with the highest n-3 FAs values for the wheat-flaxseed diet and enzyme C.

In the 2nd week (*p* = 0.003) and 10th week (*p* = 0.011), for the n-6:n-3 ratio, an interaction was recorded between the diet and enzyme sources, with the lowest ratio for the wheat-flaxseed diet and enzyme B (2nd week) and enzyme C (10th week). On the 4th (*p* ≤ 0.001), 6th (*p* ≤ 0.001), and 8th week (*p* ≤ 0.001), the diet and enzyme sources influenced the n-6:n-3 ratio, with the lowest ratio for the wheat-flaxseed diet and enzyme C.

### 3.3. Gene Expression in the Liver, Intestine, and Abdominal Fat

The influence of the corn-flaxseed and wheat-flaxseed diets with added enzymes on gene expression in the liver and abdominal fat of the laying hens is presented in Table 5. There was no interaction between the diets and the enzymes on the liver and adipocyte gene expression (*p* > 0.05), and no difference was recorded between the enzymes (*p* > 0.05). However, the corn-flaxseed diet significantly reduced the L-FABP, FADS1, ELOV2, FATP-1, and PPAR-α gene expression of the liver, and FAPB-4, PPAR-γ, and FAT gene expression in adipose tissues compared with the wheat-flaxseed diet (*p* < 0.05). The corn-flaxseed diet increased the FADS2 gene expression of the liver compared to the wheat-flaxseed diet (*p* < 0.05). 

There was no interaction between the diets and enzymes on the intestinal gene expression except for PPAR (Table 6). A wheat-flaxseed diet with enzyme C upregulated the PPAR gene expression in the duodenum.

The enzymes did not affect the intestinal gene expression (*p* > 0.05). However, the diet significantly affected the intestinal gene expression (*p* < 0.01). The corn-flaxseed diet significantly decreased FABP2, PPAR, FATP-1, and FATP-4 gene expression in the jejunum (*p* < 0.05), and decreased L-FABP and PPAR gene expression in the duodenum and FABP6V1, FATP-1, FATP-4 gene expression in the ileum (*p* < 0.01). FABP and FATP were upregulated in the intestine of the wheat-flaxseed group and downregulated in the corn-flaxseed group, where a tendency towards being an n-3 FA source rather than an n-6 FA source was observed. 

## 4. Discussion

Flaxseed is used as an n-3 source in the diet of laying hens. Many studies have shown that adding carbohydrase enzyme preparations can reduce the anti-nutritional effects of NSP and improve the utilization of flaxseed in the body [10,13]. However, the results obtained from numerous experiments are often different and there is a lack of research on proteases.

Our results showed that the addition of protease with various carbohydrase enzymes (enzyme C) had the most significant impact on weight gain in all groups. Additionally, in the 10th week, supplementing WFD with protease and cellulase (enzyme B) resulted in a considerable increase in egg weight compared to a CFD, while CFD and enzyme C showed the highest egg production during the same period. These results suggest that the digestion effect of protease alone (enzyme A) on a flaxseed diet is not as good as the combination of multiple enzymes. Overall, the combination of multiple enzymes is a better enzyme addition strategy in terms of enhancing egg production. Previous studies have also found that the addition of β-glucanase and xylanase enzymes to flaxseed diets improves egg weight and egg production in laying hens, and enhances fatty acid deposition in eggs [27].

In this study, we showed that the total n-3 deposition was enhanced in the WFD supplemented with enzyme C group, and this enzyme blend exhibited the lowest n-6:n-3 ratio. In contrast, the CFD group had higher n-6 fatty acids than the WFD group, indicating that wheat may be more suitable for the production of n-3 eggs. It is worth noting that the use of enzyme B also led to more early stage deposition of n-3 fatty acids, indicating that the combination of protease and cellulase had a positive effect on the digestion of flaxseed, and performed outstandingly in increasing egg weight. The enhancement of n-3 deposition in eggs from the wheat-flaxseed-fed group might be due to the beneficial effects of carbohydrase enzymes, like protease, amylase, cellulose, and xylanase, by breaking the bonds between sugar and NSP, releasing the nutrients for utilization by the birds [13,18]. Similar studies have shown that flaxseed alone can increase the n-3 PUFA content of eggs without significantly affecting egg production or cholesterol content, but supplementing with enzymes can have a positive effect on feed utilization, egg shell quality, and the deposition of n-3 fatty acids in eggs [8,28]. However, some studies have reported that supplementing with enzymes does not affect egg weight, α-LA, total n-3 fatty acids, total lipids, or total tocopherol content in eggs [29]. An in vitro study examined the effect of the proteinase hydrolysis of flaxseed and found that various proteinases produced different products with antioxidant properties [5]. Ferulic acid is a phenolic compound naturally present in flaxseed and has high antioxidant activities [30]. The use of carbohydrase and protease enzymes on the flaxseed byproducts increases the extraction of phenolic compounds, like ferulic acid, 10–14-fold, compared to conventional non-enzymatic extraction [31]. 

Additionally, we found that a wheat-flaxseed-based diet rich in n-3 FAs led to increased expression of L-FABP and I-FABP in the liver and intestine of laying hens, along with increased expression of PPAR-α. L-FABP is involved in FA transport and is related to the content of long-chain PUFAs [32]. I-FABP or FABP2 each have important roles in fatty acid transport and are highly expressed in the intestine after feeding vegetable oil [33,34]. PPAR-α is a transcription factor that plays a critical role in regulating lipid metabolism in the liver and other tissues. Our results suggest that a wheat-based diet promotes the absorption of n-3 and its long-chain metabolites for β-oxidation in the liver. This inference is supported by Gao [35], who investigated how L-FABP helped lipid metabolism in the chicken liver by increasing the expression of its transcriptional regulator PPAR-α. Contrary to our finding, Poirier et al. [36] reported the over-expression of L-FABP in mice given n-6 enriched sunflower oil. In this study, I-FABP was highly expressed in the wheat-flaxseed diet compared to the corn-flaxseed diet, which might have enhanced the uptake of n-3 and its long-chain metabolites to the liver for β-oxidation, and as a result, enriched the total n-3 in the egg yolk compared to the total n-6 FAs. From these results, it is clear that L-FABP and I-FABP have an affinity towards n-3 FA-enriched diets rather than n-6 FA-enriched diets. 

The main fatty acid transporter gene FATP-4 is highly expressed in enterocytes, which stimulates the uptake of long-chain FAs from the intestinal lumen to mature enterocytes [37]. In this study, the liver and intestine of laying hens fed a wheat-flaxseed diet over-expressed the fatty acid transport genes FATP-1, FATP-4, FABP-6 (V1), and FAT, which may have enhanced the uptake of C18:3n-3 and its long-chain metabolites instead of C18:2n-6 by the liver, thus enriching the eggs with n-3, DHA, ETA, and EPA. Similar to our study, the fatty acid transport genes, such as FATP-1, FATP-4, and FAT, were highly expressed in mice fed an n-3-rich fish oil diet compared with a diet rich in saturated fatty acids [38]. A recent study also found that when flaxseed and multi-carbohydrase enzymes blend were added, the wheat-fed group was found to have higher levels of growth performance and cellular pathways as well as endocrine system pathways, while the corn-fed group had significantly higher levels of avian adipose tissue by weight and inflammation [17]. This suggests that the activating effect of n-3 on fatty acid transporters is likely to be widespread. The specific activation pathway remains to be studied in future research. 

## 5. Conclusions

It is concluded that the most significant effects on the laying performances of hens are achieved by adding proteinase and several carbohydrase enzymes to a wheat-flaxseed diet. Supplementing a flaxseed diet with proteinase and cellulase enzymes can significantly increase egg weight, while using multiple carbohydrase and protease enzymes can enhance egg production. A wheat-flaxseed diet is inclined to deposit more n-3 FA and DHA than a corn-flaxseed diet, indicating possible competition between n-3 and n-6. The FABP and FATP genes were highly expressed in the wheat-flaxseed diet compared to the corn-flaxseed diet, with a tendency towards n-3 FA diets rather than n-6 FA diets.

## Figures and Tables

**Table 1:** Diet formulation and fatty acids analysis.

Ingredient %	Corn-Flaxseed Diet	Wheat-Flaxseed Diet	Flaxseed
Corn	52.00	0.00	
Wheat	0.00	65.20	
Soybean meal	27.00	13.60	
Flaxseed	10.00	10.00	
Limestone	9.00	9.00	
Calcium hydro-phosphate	1.10	0.96	
Sodium chloride	0.35	0.35	
Choline chloride	0.10	0.10	
Mineral premix ^1^	0.20	0.20	
DL-Methionine	0.17	0.18	
L-Lysine	0.00	0.33	
Vitamin premix ^2^	0.02	0.02	
Phytase	0.02	0.02	
Antioxidant	0.02	0.02	
Selenium yeast	0.02	0.02	
	100.00	100.00	
Calculated data			
MEn Poultry (mc/kg)	2.758	2.758	
Protein %	17.50	17.50	
Ca%	3.89	3.89	
NPP%	0.26	0.26	
Lysine %	1.04	1.04	
Methionine %	0.53	0.53	
M+C	0.79	0.79	
Threonine %	0.68	0.68	
	Fatty acid analysis		
Fatty acids %	Corn-flaxseed diet	Wheat-flaxseed diet	Flaxseed
Myristic acid	0.40	0.20	0.1
Palmitic acid C16:0	9.80	8.20	6.7
Margaric acid C17:0	0.40	0.30	0.1
Palmitoleic acid C16:1	0.08	0.07	0.08
Oleic acid C18:1n9c	29.02	27.08	20.5
Arachidic acid C20:0	0.14	0.08	0.09
Linoleic acid C18:2n6	35.66	33.23	14.2
Eicosadienoic acid	N.D.	N.D.	N.D.
Dihomo-γ-linolenic acid	N.D.	N.D.	N.D.
Alpha-linolenic acid n3	33.22	35.56	54.00
ETA C20:3 n3	N.D.	N.D.	N.D.
EPA C20:5 n3	N.D.	N.D.	N.D.
DHA C22:6 n3	N.D.	N.D.	N.D.

N.D. = not detected. ^1^ The mineral premix provided the following per kg of diet: Cu, 8 mg; Fe, 80 mg; Zn, 60 mg; Mn,100 mg; I, 0.35 mg; Se, 0.15 mg; ^2^ The vitamin premix provided the following per kilogram of diet: vitamin A, 10,000 IU; vitamin D3, 2400 IU; vitamin E, 20 IU; vitamin K3, 2.00 mg; thiamin, 2.00 mg; riboflavin, 6.40 mg; pyridoxine, 3.00 mg; VB12, 0.02 mg; folic acid, 1.00 mg; pantothenic acid, 10.00 mg; nicotinic acid, 30.00 mg; biotin, 0.10 mg.

**Table 2:** Sequences of primer pairs of mRNA.

mRNA	FORWARD	REVERSE
FADS1	CCGTGCCACTGTGGAGAAGATG	GCCTAGAAGCAACGCAGAGAAGAG
FADS2	TCACTTCCAACATCACGCTAAGCC	GCTGGTGGTTGTAAGGCAGGTAC
ELOV2	CACCGTCGCATACCTGCTCTG	AGGTTCTGGCACTGCAAGTTGTAG
ELOV5	GCGATGCGTCCTTATCTGTGGTG	GCTGGTCTGGAAGATTGTCAGGAC
L-FABP	GAAGAGTGTGAGATGGAGCTGCTG	GGTGATGGTGTCTCCGTTGAGTTC
PPAR-α	AGGCCAAGTTGAAAGCAGA	GTCTTCTCTGCCATGCACAA
FATP1	GTGATTCCAGAGGGCTGTGC	GGGTGGCACTCTCATTGACG
FATP4	ACAGTCGTCATCCGCAAGAAGTTC	GCCGCTCCACCTCCTGGTAG
FAT	ACCAGACCAGTAAGACCGTGAAGG	ATGTCTAGGACTCCAGCCAGTGTG
FABP2	TCAGGCTCTTGGAACCTGGAAGG	TTGGCTTCAACTCCTTCGTACACG
FABP4	ACTGAAGCAGGTGCAGAAGTGG	TGCATTCCACCAGCAGGTTCC
FABP6V1	GATCGGTCTCCCTGCTGACA	TTAGTCGTGGTGCGTCCTCC
FABP6V2	GATCGGTCTCCCTGCTGACA	TTAGTCGTGGTGCGTCCTCC
PPAR-γ	GACCTTAATTGTCGCATCCAT	CGGGAAGGACTTTATGTATGA
beta-actin	CCAGCCATGTATGTAGCCATCCAG	ACGGCCAGCCAGATCCAGAC

FADS1: fatty acid desaturase 1 encoding ∆-5 desaturase; FADS2: fatty acid desaturase 2 encoding ∆-6 desaturase; ELOV2: elongase 2; ELOV5: elongase 5; FATP1: fatty acid transport protein 1; FATP4: fatty acid transport protein 4; FABP2: fatty acid binding protein-2; FABP4: fatty acid binding protein-4; FABP6V1: fatty acid binding protein-6 variant 1; FABP6V2: fatty acid binding protein-6 variant 2; FAT: fatty acid translocase; PPAR-α: peroxisome proliferator-activated receptor alpha; PPAR-γ: peroxisome proliferator-activated receptor gamma.

**Table 3:** Effect of WFD and CFD with enzyme on the production performances of laying hens.

**Diets**	**Enzymes**	**Egg Weight (g)**						**Egg Mass (g)**						**Hen Day Egg Production (%)**					
		**0–2 Weeks**	**3–4 Weeks**	**5–6 Weeks**	**7–8 Weeks**	**9–10 Weeks**	**Total**	**0–2 Weeks**	**3–4 Weeks**	**5–6 Weeks**	**7–8 Weeks**	**9–10 Weeks**	**Total**	**0–2 Weeks**	**3–4 Weeks**	**5–6 Weeks**	**7–8 Weeks**	**9–10 Weeks**	**Total**
Corn	E-A	39.93	42.69	45.78	45.78	47.12	44.26	16.17	31.15	38.21	39.39	44.81 ^a^	33.94	40.00	72.94	83.41	85.24	90.79 ^b^	75.24
	E-B	39.86	42.83	45.60	45.87	47.25	44.28	17.06	34.95	40.78	40.09	43.31 ^ab^	35.24	41.91	81.59	89.45	87.38	91.59 ^b^	78.38
	E-C	39.43	42.65	45.37	45.59	47.98	44.21	18.03	34.88	40.56	40.09	43.57 ^ab^	35.43	45.16	81.83	89.37	87.94	94.60 ^a^	79.02
Wheat	E-A	37.97	41.93	45.29	46.35	47.93	43.90	14.25	32.58	38.69	40.61	42.39 ^b^	33.71	35.88	77.54	85.72	88.25	91.90 ^b^	75.86
	E-B	39.01	41.76	45.29	45.93	47.67	43.93	16.23	34.30	39.85	40.23	43.71 ^ab^	34.87	38.52	82.07	85.72	88.25	91.90 ^b^	75.86
	E-C	38.93	42.41	45.56	46.07	48.27	44.25	15.92	33.23	39.79	40.31	45.29 ^a^	34.91	39.68	78.58	88.02	87.46	91.67 ^b^	77.55
SEM		0.27	0.14	0.15	0.14	0.11	0.10	0.45	0.47	0.49	0.39	0.30	0.28	1.09	1.12	0.99	0.76	0.31	0.59
Main Effects																			
Corn		39.74	42.72	45.59	45.74	47.45	44.25	17.08	33.66	39.85	39.86	43.89	34.87	42.36	78.79	87.41	86.85	92.33	77.55
Wheat		38.64	42.04	45.38	46.12	47.96	44.02	15.47	33.37	39.45	40.38	43.80	34.49	38.03	79.40	87.01	87.75	92.46	76.93
E-A		39.44	42.30	45.45	45.90	47.46 ^b^	44.11	16.64	34.63 ^a^	40.32	40.16	43.51	35.05	40.22	81.83 ^a^	88.73	87.42	91.63 ^b^	77.96
E-B		39.18	42.53	45.46	45.83	48.13 ^a^	44.23	16.98	34.06 ^ab^	40.18	40.20	44.43	35.17	42.42	80.20 ^ab^	88.34	87.74	92.30 ^ab^	78.20
E-C		38.95	42.31	45.54	46.06	47.53 ^b^	44.08	15.21	31.87 ^b^	38.45	40.00	43.60	33.82	37.94	75.24 ^b^	84.57	86.75	93.25 ^a^	75.55
*p*-Value																			
Diets		0.043	0.018	0.503	0.211	0.008	0.295	0.074	0.074	0.690	0.527	0.864	0.500	0.050	0.775	0.843	0.580	0.767	0.606
Enzyme		0.752	0.745	0.965	0.798	0.008	0.831	0.230	0.230	0.255	0.978	0.334	0.104	0.241	0.043	0.186	0.877	0.019	0.146
D × E		0.504	0.474	0.647	0.755	0.470	0.677	0.812	0.812	0.820	0.841	0.014	0.978	0.920	0.331	0.630	0.648	<0.001	0.735
**Diets**	**Enzymes**	**Bodyweight Gain (g)**						**Feed Intake (g)**						**Feed Conversion Ratio FCR (%)**					
		**0–2 Weeks**	**3–4 Weeks**	**5–6 Weeks**	**7–8 Weeks**	**9–10 Weeks**	**Total**	**0–2 Weeks**	**3–4 Weeks**	**5–6 Weeks**	**7–8 Weeks**	**9–10 Weeks**	**Total**	**0–2 Weeks**	**3–4 Weeks**	**5–6 Weeks**	**7–8 Weeks**	**9–10 Weeks**	**Total**
Corn	E-A	5.30 ^a^	1.36 ^ab^	0.41	0.55	0.72	24.99	79.63	84.78	85.02	84.77 ^a^	84.95	83.83 ^a^	4.49	2.46	2.11	2.11	1.96 ^ab^	2.38
	E-B	4.64 ^a^	2.36 ^a^	0.39	1.35	0.38	27.38	79.26	84.40	84.75	84.40 ^ab^	84.82	83.53 ^ab^	5.01	2.74	2.23	2.12	1.95 ^ab^	2.37
	E-C	4.59 ^a^	0.86 ^b^	0.59	0.4	0.64	21.26	79.87	84.82	84.95	84.37 ^ab^	84.87	83.77 ^a^	4.76	2.43	2.08	2.15	1.89 ^b^	2.47
Wheat	E-A	2.77 ^b^	2.20 ^a^	0.66	1.55	0.58	23.29	76.12	82.55	82.65	83.90 ^b^	84.95	82.03 ^d^	4.86	2.49	2.09	2.09	1.88 ^b^	2.35
	E-B	5.60 ^a^	0.86 ^b^	0.44	0.56	0.83	24.87	78.10	84.07	83.60	84.45 ^ab^	84.87	83.02 ^bc^	5.73	2.60	2.19	2.09	2.02 ^a^	2.47
	E-C	2.26 ^b^	2.11 ^a^	1.28	0.84	0.43	20.75	76.68	83.80	82.77	84.55 ^ab^	84.90	82.54 ^cd^	4.83	2.45	2.08	2.11	1.94 ^ab^	2.37
SEM		0.30	0.17	0.11	0.15	0.09	0.82	0.36	0.20	0.24	0.09	0.03	0.15	0.15	0.04	0.03	0.02	0.02	0.02
Main Effects																			
Corn		4.84	1.53	0.47	0.77	0.58	24.54	79.59	84.67	84.91	84.51	84.88	83.71	4.75	2.54	2.14	2.12	1.93	2.41
Wheat		3.54	1.72	0.80	0.98	0.61	22.97	76.97	83.47	83.01	84.30	84.91	82.53	5.14	2.51	2.12	2.10	1.94	2.40
E-A		4.03 ^ab^	1.78	0.54	1.05	0.65	24.14 ^ab^	77.88	83.67	83.84	84.33	84.95	82.93	4.68	2.47 ^b^	2.10	2.10	1.91	2.36
E-B		5.12 ^a^	1.61	0.42	0.96	0.61	26.13 ^a^	78.68	84.23	84.18	84.43	84.84	83.27	5.37	2.67 ^a^	2.21	2.12	1.96	2.47
E-C		3.43 ^b^	1.48	0.94	0.62	0.53	21.01 ^b^	78.28	84.31	83.86	84.46	84.88	83.16	4.80	2.44 ^b^	2.08	2.11	1.95	2.37
*p*-Value																			
Diets		0.007	0.516	0.119	0.465	0.836	0.325	<0.001	0.001	<0.001	0.242	0.673	<0.001	0.177	0.686	0.727	0.561	0.733	0.812
Enzyme		0.015	0.717	0.110	0.468	0.862	0.040	0.502	0.208	0.695	0.838	0.402	0.391	0.120	0.034	0.206	0.947	0.441	0.069
D × E		0.005	0.001	0.430	0.052	0.260	0.872	0.192	0.059	0.341	0.044	0.950	0.047	0.652	0.575	0.970	0.851	0.030	0.978

The superscripts a, b, c, and d represents significant difference between treatments. Significance was set at <0.05. n = 6. E-A= enzyme A, E-B = enzyme B, E-C = enzyme C. Enzyme A contains neutral protease 10,000 U/g, and alkaline protease 40,000 U/g. Enzyme B contains neutral protease 10,000 U/g, alkaline protease 40,000 U/g, and cellulase 4000 U/g. Enzyme C contains neutral protease 10,000 U/g, xylanase 35,000 U/g, β-mannanase 1500 U/g, β-glucanase 2000 U/g, cellulose 500 U/g, amylase 100 U/g, and pectinase 10,000 U/g.

**Table 4:** Effect of flaxseed with enzymes on the FA profile of egg yolk expressed as mg/g of egg yolk.

F.A	Weeks	Means						SEM	Main Effects					*p*-Value		
		Corn E-A	Corn E-B	Corn E-C	Wheat E-A	Wheat E-B	Wheat E-C		Corn	Wheat	E-A	E-B	E-C	Diet	Enzyme	D × E
DHA	2nd	25.43 ^c^	30.16 ^b^	29.20 ^b^	32.81 ^a^	32.52 ^a^	32.17 ^a^	0.49	28.26	32.50	29.12 ^b^	31.34 ^a^	30.68 ^a^	<0.001	0.003	<0.001
	4th	37.24	36.51	36.65	41.38	41.89	41.58	0.48	36.80	41.62	39.31	39.20	39.11	<0.001	0.959	0.665
	6th	40.62	52.19	46.36	51.72	61.18	49.63	1.21	46.39	54.18	46.17 ^b^	56.68 ^a^	47.99 ^b^	<0.001	<0.001	0.053
	8th	61.33	63.27	62.74	67.38	67.40	64.23	0.61	62.44	66.34	64.35	65.33	63.48	<0.001	0.345	0.202
	10th	60.57 ^c^	69.16 ^b^	64.80 ^bc^	76.70 ^a^	83.51 ^a^	68.73 ^b^	1.57	64.84	76.31	68.63 ^b^	76.34 ^a^	66.76 ^b^	<0.001	0.001	0.037
Total n-6	2nd	337.85 ^a^	329.41 ^b^	329.25 ^b^	316.76 ^d^	326.54 ^bc^	320.73 ^cd^	1.51	332.17	321.34	327.31	327.97	324.99	<0.001	0.498	0.005
	4th	507.65 ^ab^	489.20 ^b^	522.67 ^a^	449.89 ^c^	448.47 ^c^	447.10 ^c^	5.75	506.51	448.49	478.77	468.83	484.89	<0.001	0.067	0.046
	6th	652.13	650.86	651.62	545.92	550.67	543.78	9.10	651.54	546.79	599.03	600.76	597.70	<0.001	0.859	0.771
	8th	816.19	784.12	813.09	653.40	651.72	655.58	13.94	804.46	653.57	734.79	717.92	734.34	<0.001	0.412	0.528
	10th	864.44 ^a^	790.29 ^b^	858.48 ^a^	652.16 ^c^	652.22 ^c^	651.34 ^c^	16.56	837.74	651.91	758.30 ^a^	721.25 ^b^	754.92 ^a^	<0.001	<0.001	<0.001
Total n-3	2nd	188.13	192.91	192.13	204.58	204.78	197.88	1.36	191.06	202.41	196.35	198.84	195.00	<0.001	0.235	0.071
	4th	207.02	233.19	221.62	198.61	249.19	219.91	3.58	220.61	222.57	202.81 ^c^	241.19 ^a^	220.76 ^b^	0.688	<0.001	0.122
	6th	289.72	333.65	300.49	332.20	396.37	361.65	6.60	307.95	363.40	310.96 ^c^	365.01 ^a^	331.07 ^b^	<0.001	<0.001	0.279
	8th	386.07 ^d^	410.80 ^c^	387.34 ^d^	440.62 ^b^	506.66 ^a^	435.88 ^b^	7.04	394.74	461.05	413.35 ^b^	458.73 ^a^	411.61 ^c^	<0.001	<0.001	<0.001
	10th	392.41 ^d^	418.44 ^c^	382.92 ^d^	452.43 ^b^	511.37 ^a^	440.87 ^b^	7.42	397.9	468.22	422.42 ^b^	464.90 ^a^	411.89 ^c^	<0.001	<0.001	<0.001
n-6:n-3	2nd	1.80 ^a^	1.71 ^b^	1.71 ^b^	1.55 ^d^	1.60 ^cd^	1.62 ^c^	0.02	1.74	1.59	1.67	1.65	1.67	<0.001	0.665	0.003
	4th	2.45	2.10	2.36	2.27	1.80	2.07	0.04	2.30	2.05	2.36 ^a^	1.95 ^c^	2.21 ^b^	<0.001	<0.001	0.610
	6th	2.25	1.96	2.18	1.65	1.39	1.51	0.06	2.13	1.52	1.95 ^a^	1.67 ^c^	1.84 ^b^	<0.001	<0.001	0.427
	8th	2.11	1.91	2.10	1.49	1.29	1.51	0.06	2.04	1.43	1.80 ^a^	1.60 ^b^	1.80 ^a^	<0.001	<0.001	0.920
	10th	2.21 ^a^	1.89 ^b^	2.25 ^a^	1.44 ^c^	1.28 ^d^	1.48 ^c^	0.06	2.11	1.40	1.82 ^a^	1.58 ^b^	1.86 ^a^	<0.001	<0.001	0.011

FA = fatty acids. The superscripts a, b, c, and d represents significant difference between treatments. Significance was set at < 0.05. n = 6. E-A = enzyme A, E-B = enzyme B, E-C = enzyme C. Enzyme A contains neutral protease 10,000 U/g, and alkaline protease 40,000 U/g. Enzyme B contains neutral protease 10,000 U/g, alkaline protease 40,000 U/g, and cellulase 4000 U/g. Enzyme C contains neutral protease 10,000 U/g, xylanase 35,000 U/g, β-mannanase 1500 U/g, β-glucanase 2000 U/g, cellulose 500 U/g, amylase 100 U/g, and pectinase 10,000 U/g.

**Table 5:** Effects of WFD and CFD with enzymes on liver and adipocytes relative gene expression.

Tissue	Gene Exp.	Means						SEM		Main Effects					*p*-Value	
		Corn E-A	Corn E-B	Corn E-C	Wheat E-A	Wheat E-B	Wheat E-C		Corn	Wheat	E-A	E-B	E-C	Diet	Enzyme	D × E
	L-FABP	1.15	1.13	1.18	1.58	1.54	1.68	0.08	1.15	1.60	1.36	1.34	1.43	0.006	0.869	0.967
Liver	FADS1	1.10	1.08	1.12	1.57	1.71	1.59	0.06	1.10	1.62	1.33	1.39	1.36	<0.001	0.816	0.619
	FADS2	2.71	2.64	2.70	1.58	1.39	1.34	0.14	2.68	1.44	2.15	2.02	2.02	<0.001	0.846	0.908
	ELOV2	0.95	0.75	0.85	2.21	2.01	2.21	0.15	0.85	2.15	1.58	1.38	1.53	<0.001	0.710	0.978
	ELOV5	2.08	2.13	1.41	1.99	1.94	1.88	0.17	1.88	1.94	2.04	2.04	1.64	0.866	0.585	0.717
	FATP-1	0.88	0.73	0.86	1.41	1.67	1.50	0.09	0.82	1.52	1.14	1.20	1.18	<0.001	0.959	0.552
	PPAR-α	0.56	0.49	0.48	1.20	1.36	1.20	0.07	0.51	1.26	0.85	0.93	0.84	<0.001	0.620	0.413
	FABP4	1.26	1.25	1.26	2.24	2.37	2.38	0.12	1.26	2.33	1.75	1.81	1.82	<0.001	0.930	0.911
Adipocytes	PPARγ	0.93	0.98	0.93	1.58	1.70	1.55	0.09	0.95	1.61	1.25	1.34	1.24	<0.001	0.828	0.963
	FATP1	1.53	1.54	1.59	1.54	1.52	1.57	0.03	1.56	1.55	1.54	1.53	1.58	0.863	0.774	0.981
	FATP4	1.31	1.35	1.34	1.31	1.37	1.34	0.02	1.33	1.34	1.31	1.36	1.34	0.898	0.634	0.964
	FAT	1.23	1.30	1.20	1.58	1.62	1.51	0.04	1.24	1.57	1.40	1.46	1.36	<0.001	0.475	0.974

FADS1: fatty acid desaturase 1 encoding ∆-5 desaturase; FADS2: fatty acid desaturase 2 encoding ∆-6 desaturase; ELOV-2: elongase 2; ELOV-5: elongase 5; FATP1: fatty acid transport protein 1; FATP-4: fatty acid transport protein-4; FABP2: fatty acid binding protein-2; FABP4: fatty acid binding protein-4; FAT: fatty acid translocase; PPAR-α: peroxisome proliferator-activated receptor alpha; PPAR-γ: peroxisome proliferator-activated receptor gamma. Significance was set at <0.05. n = 6. E-A = enzyme A, E-B = enzyme B, E-C = enzyme C. Enzyme A contains neutral protease 10,000 U/g, and alkaline protease 40,000 U/g. Enzyme B contains neutral protease 10,000 U/g, alkaline protease 40,000 U/g, and cellulase 4000 U/g. Enzyme C contains neutral protease 10,000 U/g, xylanase 35,000 U/g, β-mannanase 1500 U/g, β-glucanase 2000 U/g, cellulose 500 U/g, amylase 100 U/g, and pectinase 10,000 U/g.

**Table 6:** Effects of WFD and CFD with enzymes on intestinal gene expression.

Tissue	Gene Exp.	Means						SEM	Main Effects					*p*-Value		
		Corn E-A	Corn E-B	Corn E-C	Wheat E-A	Wheat E-B	Wheat E-C		Corn	Wheat	E-A	E-B	E-C	Diet	Enzyme	D × E
	L-FABP	1.36	1.36	1.36	1.40	1.50	1.36	0.02	1.36	1.42	1.38	1.43	1.36	0.22	0.106	0.309
Jejunum	FABP2	1.24	1.23	1.23	1.68	1.62	1.55	0.04	1.23	1.61	1.46	1.42	1.39	<0.001	0.586	0.638
	FABP6V1	1.23	1.30	1.24	1.21	1.24	1.22	0.04	1.26	1.22	1.22	1.27	1.23	0.710	0.870	0.970
	FABP6V2	1.30	1.34	1.28	1.36	1.47	1.48	0.06	1.31	1.44	1.33	1.41	1.38	0.270	0.870	0.880
	PPAR-α	0.58	0.60	0.58	1.01	1.13	1.40	0.07	0.59	1.18	0.80	0.87	0.99	<0.001	0.231	0.209
	FATP-1	1.00	1.18	0.90	1.21	1.46	1.36	0.06	1.02	1.34	1.10	1.32	1.13	0.006	0.214	0.609
	FATP4	0.96	1.10	1.10	1.54	1.12	1.45	0.06	1.05	1.37	1.25	1.11	1.27	0.009	0.439	0.132
Duodenum	L-FABP	1.27	1.25	1.27	1.70	1.69	1.55	0.04	1.27	1.65	1.48	1.47	1.41	<0.001	0.466	0.355
	FABP2	1.73	1.76	1.74	1.71	1.78	1.71	0.03	1.74	1.74	1.72	1.77	1.73	0.932	0.757	0.926
	FABP6V1	0.86	0.91	0.94	1.02	0.92	1.04	0.05	0.90	0.99	0.94	0.92	0.99	0.411	0.842	0.859
	FABP6V2	1.67	1.59	1.51	1.36	1.47	1.48	0.06	1.59	1.44	1.52	1.53	1.50	0.230	0.970	0.640
	PPAR-α	0.51 ^cd^	0.38 ^d^	0.28 ^d^	0.89 ^b^	0.80 ^bc^	1.30 ^a^	0.07	0.40	1.00	0.70	0.59	0.79	0.000	0.245	0.015
	FATP-1	0.73	0.99	0.71	0.88	0.95	1.07	0.06	0.81	0.97	0.81	0.97	0.89	0.158	0.466	0.345
	FATP4	1.04	1.21	1.32	0.85	1.11	1.01	0.06	1.19	0.99	0.95	1.16	1.17	0.100	0.233	0.765
Ileum	L-FABP	1.46	2.10	1.80	1.43	1.49	1.50	0.79	1.78	1.47	1.44	1.79	1.65	0.260	0.570	0.670
	FABP2	0.84	1.66	1.00	0.89	0.77	1.11	0.11	1.36	1.42	1.38	1.43	1.36	0.220	0.106	0.309
	FABP6V1	0.51	0.31	0.29	0.79	0.80	1.07	0.07	1.23	1.61	1.46	1.42	1.39	<0.001	0.586	0.638
	FABP6V2	1.56	1.28	0.86	1.15	1.24	1.04	0.10	1.26	1.22	1.22	1.27	1.23	0.710	0.870	0.970
	PPAR-α	1.50	1.57	1.42	2.09	1.85	1.94	0.08	1.31	1.44	1.33	1.41	1.38	0.270	0.870	0.880
	FATP-1	0.92	1.05	0.83	0.92	0.93	1.01	0.00	0.59	1.18	0.80	0.87	0.99	<0.001	0.231	0.209
	FATP4	1.06	1.01	1.03	1.41	1.48	1.46	0.06	1.02	1.34	1.10	1.32	1.13	0.006	0.214	0.609

The superscripts a, b, c, and d represents significant difference between treatments. FATP1: fatty acid transport protein 1; FATP4: fatty acid transport protein-4; FABP2: fatty acid binding protein-2; FABP4: fatty acid binding protein-4; FABP6V1: fatty acid binding protein-6 variant 1, FABP6V2: fatty acid binding protein-6 variant 2, FAT: fatty acid translocase; PPAR-α: peroxisome proliferator-activated receptor alpha; PPAR-γ: peroxisome proliferator-activated receptor gamma. Significance was set at <0.05. E-A = enzyme A, E-B = enzyme B, E-C = enzyme C. Enzyme A contains neutral protease 10,000 U/g, and alkaline protease 40,000 U/g. Enzyme B contains neutral protease 10,000 U/g, alkaline protease 40,000 U/g, and cellulase 4000 U/g. Enzyme C contains neutral protease 10,000 U/g, xylanase 35,000 U/g, β-mannanase 1500 U/g, β-glucanase 2000 U/g, cellulose 500 U/g, amylase 100 U/g, and pectinase 10,000 U/g.

## Data Availability

Data are contained within the article.
